# Rationale and design of a randomised controlled trial evaluating the effectiveness of an exercise program to improve the quality of life of patients with heart failure in primary care: The EFICAR study protocol

**DOI:** 10.1186/1471-2458-10-33

**Published:** 2010-01-25

**Authors:** Ana Zuazagoitia, Gonzalo Grandes, Jesús Torcal, Iñaki Lekuona, Pilar Echevarria, Manuel A Gómez, Mar Domingo, Maria M de la Torre, Jose I Ramírez, Imanol Montoya, Juana Oyanguren, Ricardo Ortega-Sánchez Pinilla

**Affiliations:** 1 Primary Care Research Unit of Bizkaia, Basque Health Service-Osakidetza , CAIBER, Bilbao, Spain; 2 Basauri-Ariz Health Centre, Basque Health Service - Osakidetza, Bizkaia, Spain; 3 Cardiology Service, Galdakao Hospital, Basque Health Service - Osakidetza, Bizkaia, Spain; 4 Galdakao Health Centre, Basque Health Service - Osakidetza, Bizkaia, Spain; 5 La Alamedilla Health Centre, Castilla y León Health Service - SACYL, Salamanca, Spain; 6 Sant Roc Health Centre. Catalan Health Service - ICS. Barcelona. Spain; 7 Casa de Barco Health Centre Castilla y León Health Service - SACYL, Valladolid, Spain; 8 Calviá Health Centre. Balearic Islands Health Service - IBSALUT, Mallorca, Spain; 9 Santa Barbara Health Centre, Castilla-La Mancha Health Service - SESCAM, Toledo, Spain; 10 redIAPP: Red de Investigación en Actividades Preventivas y Promoción de la Salud, Spain

## Abstract

**Background:**

Quality of life (QoL) decreases as heart failure worsens, which is one of the greatest worries of these patients. Physical exercise has been shown to be safe for people with heart failure. Previous studies have tested heterogeneous exercise programs using different QoL instruments and reported inconsistent effects on QoL. The aim of this study is to evaluate the effectiveness of a new exercise program for people with heart failure (EFICAR), additional to the recommended optimal treatment in primary care, to improve QoL, functional capacity and control of cardiovascular risk factors.

**Methods/Design:**

Multicenter clinical trial in which 600 patients with heart failure in NYHA class II-IV will be randomized to two parallel groups: EFICAR and control. After being recruited, through the reference cardiology services, in six health centres from the Spanish Primary Care Prevention and Health Promotion Research Network (*redIAPP*), patients are followed for 1 year after the beginning of the intervention. Both groups receive the optimized treatment according to the European Society of Cardiology guidelines. In addition, the EFICAR group performs a 3 month supervised progressive exercise program with an aerobic (high-intensity intervals) and a strength component; and the programme continues linked with community resources for 9 months. The main outcome measure is the change in health-related QoL measured by the SF-36 and the Minnesota Living with Heart Failure Questionnaires at baseline, 3, 6 and 12 months. Secondary outcomes considered are changes in functional capacity measured by the 6-Minute Walking Test, cardiac structure (B-type natriuretic peptides), muscle strength and body composition. Both groups will be compared on an intention to treat basis, using multi-level longitudinal mixed models. Sex, age, social class, co-morbidity and cardiovascular risk factors will be considered as potential confounding and predictor variables.

**Discussion:**

A key challenges of this study is to guarantee the safety of the patients; however, the current scientific evidence supports the notion of there being no increase in the risk of decompensation, cardiac events, hospitalizations and deaths associated with exercise, but rather the opposite. Safety assurance will be based on an optimized standardised pharmacological therapy and health education for all the participants.

**Trial Registration:**

Clinical Trials.gov Identifier: NCT01033591

## Background

Heart failure (HF) affects more than 15 million people out of the 900 million who live in the 51 countries represented in the European Society of Cardiology [1]. In Spain it occurs in 1-2% of individuals older than 40 years old and in 10% of those over 60 [2]. It is also the first cause of hospital admission and involves a yearly expenditure of four thousand million Euros for health services [3]. Although survival rates of people with HF have increased with the current pharmacological treatments, this does not necessarily entail an improvement in quality of life [4]. Quality of life worsens with increasing severity of HF. This is one of the greatest concerns of such patients and some studies suggest that it is a predictive variable for the progress of disease, independently of other prognostic factors, such as left ventricular ejection fraction [5,6]. Non-pharmacological interventions, such as physical exercise may have a great impact on the quality of life, but this remains poorly studied. In particular, the studies carried out have used very heterogeneous exercise programmes, have evaluated quality of life in very different ways and have reported inconsistent results [7].

Among the current recommendations, there are no clear guidelines about the type, intensity, duration and progress of exercise programmes that should be carried out by people with HF [8,9]. Also, the minimum exercise required still remains unknown, let alone what are the optimum conditions. For this reason primary care doctors only give these patients the general advice of walking at least 30 minutes per day, preferably every day of the week [10]. Reviewing the opinion of various authors and looking at recent studies that advocate high-intensity interval training, the ideal would be to evaluate an exercise programme that covers the following aspects: (1) short bursts of high-intensity aerobic exercise; (2) exercise combined with building strength/stamina; (3) supervised initially in the health centre, for the intervention to be adapted to each patient and to guarantee adherence; and (4) linked to resources external to the health service in the community or in the home of the patient, to achieve long-term continuity [11-15]. An intervention of this type would be innovative and could be highly effective, given that it brings together the scientific evidence of previous interventions that have been shown to be hypothetically effective.

On the other hand, physical exercise per se has been shown to be a safe intervention for people suffering from HF. This assertion has been supported by the recent HF-Action study, the largest clinical trial carried out on 2331 people with HF in NYHA class II-IV, to evaluate the effect of aerobic exercise in such patients [16,17]. The study concluded that exercise has a small beneficial effect on mortality and hospital admissions. Based on this study, it is plausible to hypothesise that the great contribution of exercise may be reflected in functional capacity and quality of life. In this study, the "state of health" (quality of life, symptoms, physical and social limitations) greatly improved in only three months. At the time the difference between the groups for comparison was not considered to be clinically relevant and only a small effect, of less than two points, could be attributed to exercise. In subsequent follow-ups, this limited difference remained constant, throughout the three years of monitoring [16]. The authors admitted that the lack of further improvement in scores after three months may have been a reflection of the exercise intervention not having been optimal, and the fact that the long-term adherence to the exercise programme was low and that the usual care group increased its physical activity.

## Objectives

To evaluate the effectiveness of a progressive exercise programme (high-intensity interval + strength training), in addition to optimal pharmacological and non-pharmacological treatments, to improve the health-related quality of life of those suffering from class II-IV heart failure, their functional capacity and prognosis.

### Main objective

- To estimate the improvement of health-related quality of life in patients involved in the EFICAR programme and the control group, and also any difference between the two groups in terms of improvement in quality of life, which would be an effect that could be attributed to exercise.

### Secondary objectives

- To estimate the improvement in functional capacity, control of risk factors and prognostic factors in the EFICAR and in the control group, and also any difference between the two groups, that could be attributed to exercise.

- To estimate any changes in the effect of exercise as a function of the various subgroups of age, sex, risk factors and co- morbidity.

## Methods/Design

### Study design

This is a multicenter, randomized, controlled clinical trial, in which people suffering from Heart Failure (HF) are assigned to two parallel groups: the EFICAR group (exercise + optimised usual care) and control group (only optimised usual care).

**Figure 1:**
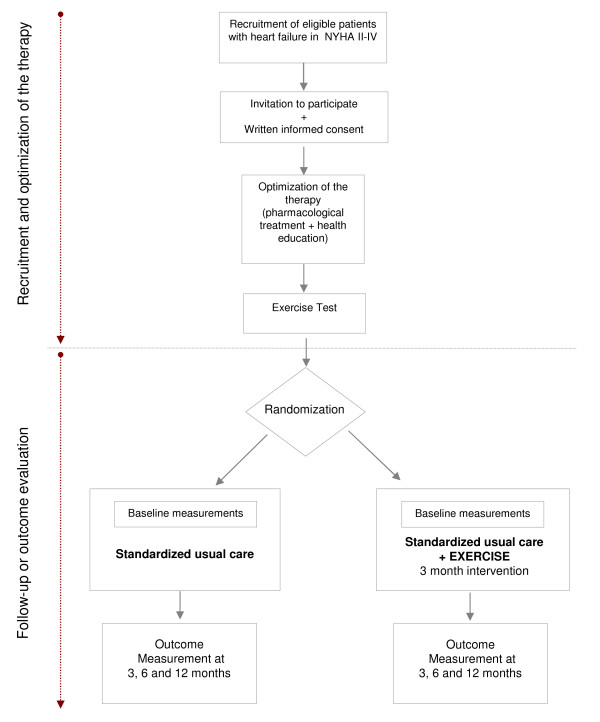
**Algorithm of the study**.

The candidates to be included in the study go through two consecutive phases: 1^st ^phase of "therapy optimisation" prior to inclusion in the study, and 2^nd ^phase of "monitoring and evaluation of results". Both groups are subject to a series of standardised elements of care: optimised pharmacological treatment, health education and support for the development of self-care. Patients are monitored for 3, 6 and 12 months. Blind measurements of quality of life, functional capacity and risk factors are repeated three times (see Figure 1).

### Settings

Six primary health centres, part of the Spanish Primary Care Prevention and Health Promotion Research Network (*redIAPP*), henceforth referred to as the redIAPP network, were selected, accredited and financially supported the Carlos III Health Institute:

- Basauri-Ariz Health Centre. Basque Health Service - Osakidetza. Bizkaia. Spain.

- Galdakao Health Centre. Basque Health Service - Osakidetza. Bizkaia. Spain.

- La Alamedilla Health Centre. Castilla y León Health Service. SACYL. Salamanca. Spain.

- Sant Roc Health Centre. ICS. Catalan Health Service. Barcelona. Spain.

- Casa de Barco Health Centre. Castilla y León Health Service. SACYL. Valladolid. Spain.

- Calviá Health Centre. Balearic Islands Health Services. IBSALUT. Mallorca. Spain.

Each collaborating centre has an established logistical structure, derived from the undertaking of the "Multicentric Assessment of Experimental Programme of Physical Activity (PEPAF)" research project. This structure is composed of:

- A laboratory for measuring physical health with various cycleergometers and hear rate monitors.

- Shared databases.

In addition, the whole redIAPP network has access to an Integrated Database Management System based on a virtual private network which links all the collaborating centres.

The Primary Care Research Unit of Bizkaia, the management centre for the design and analysis of this study, has:

- A user license for SAS statistical package with which all the analyses are carried out.

- Local ICT Infrastructure to undertake training activities and coordination meetings.

- Technical secretarial support.

### Study population

#### Inclusion criteria

• Age ≥ 18 years.

• Diagnosis of HF on the basis of signs and symptoms (Framingham criteria) and evidence of structural heart alterations detected by echocardiography (Echo). Echo scanning guarantees that we are dealing with patients suffering from HF avoiding confounding clinical factors.

• Left ventricle ejection fraction < 45%.

• NYHA functional class II-IV, or Stages B and C of the American Heart Association, in a stable situation for at least the previous four weeks, with no changes in baseline functional status, no signs of congestion or changes in weight faster than 2 kg in three days.

• Receiving optimal treatment with angiotensin converting enzyme inhibitors (ACEI), angiotensin II receptor antagonists (ARA-II), beta blockers, diuretics, and aldosterone antagonists at stable doses for the previous four weeks, as long as there are no justified contraindications for their use, and meeting the clinical practice guidelines of the European Society of Cardiology [1].

• Anticoagulated patients without atrial fibrillation, ejection fraction < 30%, presence of intracardiac thrombi, or history of embolism.

• In cases of sinusal rhythm or atrial fibrillation, ventricular response is under control both at rest and during exercise (90 beats/minute at rest, and 130 beats/minute during moderate exercise).

• Absence of arrhythmia in exercise stress test that would contraindicate exercise.

• Able to attend an exercise programme and travel to the reference laboratory.

• Informed consent confirmed in writing.

#### Exclusion criteria

• Physical and mental comorbidity which preventes undertaking the exercise programme.

• Major cardiovascular events (in the previous 6 weeks) or cardiovascular procedures, including cardiac resynchronization or implantation of a defibrillator.

• Heart failure pending intervention (mitral valve replacement/repair, ventricular reconstruction, pacemaker/resynchronization pacemaker, implantable defibrillator, transplant), given that the procedures greatly change the baseline functional status and the prognosis of the disease.

• Heart failure secondary to congenital heart disease or hypertrophic obstructive cardiomyopathy, given that there is a formal contraindication for exercise in such clinical situations.

• Carrier of a fixed-rate pacemaker.

• Exercise test that contraindicates exercise for safety reasons, especially in the case of exercise-induced arrhythmia.

• Poor cognitive state, depression or psychiatric disorder that prevent adherence to an exercise programme.

• Inability to travelling to the health centre by their own means.

### Recruiting

To recruit patients, the nurse sets up an active surveillance system to identify new HF patients in the health centre. The principal investigator informs the patient about the study, invites them to participate, provides the consent form, makes an initial evaluation, carries out an electrocardiogram (ECG) and registers the information.

### Optimisation of the common treatment for both groups in the study

Once the patient has been recruited, a trained nurse carries out the "optimisation of the treatment". An "information visit" is conducted in which an ECG and a blood sample are requested, and the results of these tests are assessed at the "first medical appointment" with the principal investigator of the health centre. If the patient is "stable" (see discussion) a new "follow up appointment" is scheduled after four weeks with a new prior blood sample. During the stabilisation period, the patient attends five appointments to evaluate prognostic clinical indicators, the battery of health education activities and compliance with non-pharmacological measures. This stabilisation phase lasts for four to six weeks prior to randomisation.

Assessments are made of the patients' cardiac baseline prior to randomisation. In line with this, when the patient is clinically stable, an appointment is made the cardiologist to carry out an exercise stress test, with an echo study and Holter monitor (if clinically required) and the outcome variables are measured.

### Randomisation

This is carried out centrally, once all baseline measurements are taken, by phoning to the Primary Care Research Unit of Bizkaia, where the patient is registered and assigned to one of the two study groups in 1:1 ratio by block randomisation of n patients and stratified by health centres.

### Protocol for the control group

"Optimal therapeutic monitoring appointments" are established at 4, 8, 12, 24 and 48 weeks (1 year), in which clinical assessments made in the first appointment are repeated. In such visits follow-up ECG and blood samples are also required.

### Protocol for the intervention group

The EFICAR group differs from the control group only in terms of the exercise programme, which has two phases. The exercise programme is a combination of "high-intensity interval training" and "muscular strength training". It has been demonstrated that high intensity aerobic exercise leads to better aerobic and cardiovascular changes than light/moderate exercise in patients with HF [13,14].

**Figure 2:**
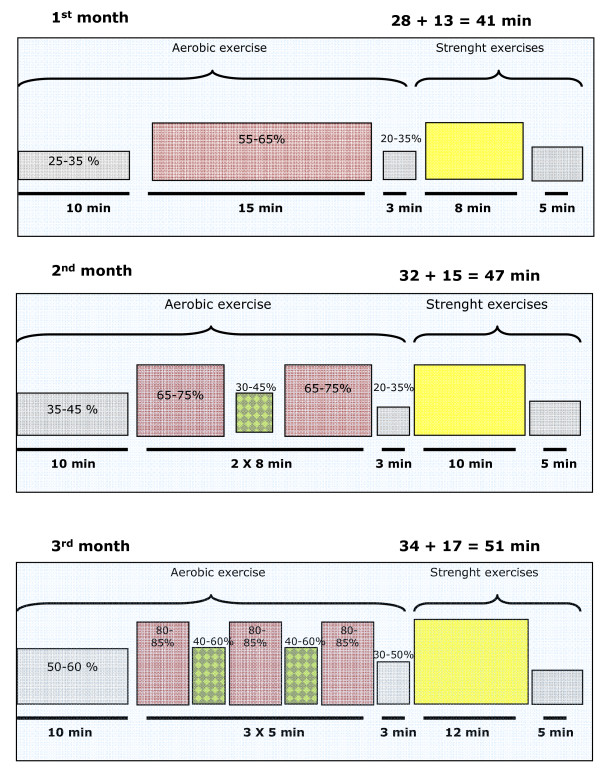
**Exercise program for the EFICAR group**.

• *1st PHASE *(12 weeks, 3 sessions/week): for the first three months, patients carry out a progressive exercise programme of 36 sessions under the supervision of the nurse, starting at low intensity (first month) and increasing it month by month (See Figure 2).

Aerobic training: Aerobic exercise is undertaken on a cycloergometer, including warm-up, conditioning and cool down phase. From the beginning, the exercise sessions are personalised according to the physical condition of each patient. The first month is for "start-up", the second for "progress" and the third for "maintenance". For those patients that are very weak or are not used to aerobic exercise, the first sessions are of light-to-moderate intensity [9,18].

The Borg scale is used to evaluate the "perception of effort" during and after each session (values ranging between 6 and 20). This is a very useful scale to determine intensity with beta blockers, auricular fibrillation, pace-makers or other conditions which may alter the normal response of the heart rate to exercise [19]. The exercise is also monitored with respect to the emergence of symptoms. Aerobic exercise is done before the strength training to guarantee activation of the muscles and cardiovascular system.

Muscular strength training: muscular strength work is a essential aspect of the rehabilitation of patients with HF [15]. To avoid training-related increase of the hemodynamic load the exercise is isotonic. In each session, after 5 min of stretching and 8-12 min of aerobic exercise, six types of exercise are carried out to work various muscle groups: biceps, upper arms and shoulders, knee extensors and flexors, hip flexors and extensors, and plantar flexors. For muscular strength training, weights are used for upper body and resistance bands for the lower body. A different percentage of body weight is applied for each muscular group. Each patient is regularly informed of the progress they have made.

• *2nd PHASE*: The training given in the first phase is intended to ensure that the patients make this exercise part of their routine. They are trained to carry out a type of interval aerobic exercise to they can keep up in their own environment, indefinitely and independently, together with muscular strength exercises. In addition, they are taught how to self-regulate the exercise intensity (Borg Scale, pulsometers, symptoms).

### Outcome measures

These measurements are made by an interviewer blind to the group the patient has been assigned to.

*Primary outcome measure *(basal, 3, 6 and 12 months):

• Changes in health-related quality of life (HRQL) (SF36, Minnesota Living with Heart Failure Questionnaire - MLHFQ).

The HRQL questionnaire is measured blindly with self-administered questionnaires: the Spanish version of the SF-36 and MLHFQ. Both questionnaires will be filled out before and after the intervention. SF-36 generates an 8-dimension health profile and two summary scores for the physical and mental components. The 21 items from the MLHFQ record the perception of the patient in relation to how the HF affects the emotional, social, physical and mental dimensions of their HRQL. Both questionnaires have been validated in the Spanish population [20,21].

*Secondary outcome measures *(basal, 3, 6 and 12 months):

• Functional capacity, measured with the 6-minute Walking Test [22].

• Muscular strength, measured by dynamometry [23].

• Body composition (body fat and muscle percentage) [23].

• Structural changes in the heart (natriuretic peptide levels, prognostic factor) [24].

*Predictor or Confounding variables*: sex, age, comorbidity and risk factors.

### Adverse events

Provisional analysis of the data is performed by an independent committee to monitor safety. This committee will define the indicators in relation to mortality, hospital admissions and cardiac decompensation events, having independent access to the necessary data. The committee will be composed of people who are independent of the body in charge of the management of the study and of the researchers and will be blind to the assignment of patients to the comparison groups, will review the data every quarter and will make recommendations to the team directing the project with respect to the continuation or the interruption of the study.

### Follow up period

One year since the beginning of the intervention.

### Sample size

A total of 600 patients will be recruited, 300 per group. This simple size gives a statistical power of higher than 80%, to enable any difference between the control and the intervention group greater than 3 with respect to the primary outcome scale from the physical functioning SF-36 and MLHQ to be found to be significant, with a significance level of 5% using the two-tailed Student's t test (standard deviations obtained from prior studies with similar population to that of our project: SD = 10 points of the quality of life questionnaires SF-36 and MLHQ) [12,25,26]. Given other reports, it can be expected that the losses during follow-up will be around 20%, the correlation between the measurements of the same individual 0.6 and the correlation intracentre 0.01.

### Statistical analysis

The analysis will be focussed on demonstrating, by analysis of covariance, any improvements at each follow-up, comparing the two groups, adjusted for the baseline level. The effect attributable to the intervention will be estimated by comparing differences in degree of improvement between the groups and the 95% confidence intervals will be calculated. The comparisons will be adjusted for potential confounding factors, by stratified analysis and statistical models, linear for continuous variable outcomes and logistic for dychotomic variables. In order to analyse the overall evolution in the course the one year of monitoring, the time effect on three repeated measures taken for each subject will be estimated, using mixed linear regression models with fixed effects (time, intervention, interaction between time and intervention) and randomised (specific effect of each subject and centre on the baseline level and the effect of time). These models will take into account the longitudinal structure of the three repeated measurements, as well as the hierarchical and multicentre structure of the data. These models will be extended to adjust for hypothetical prediction and modification variables of effect: age, sex, risk factors and comorbidity. All the analysis will be carried out using the SAS statistical package.

### Quality control

Various processes were undertaken in order to guarantee the quality of the data of the study, and thus maximise the validity and reliability of the programme and measurement of outcomes. These are:

• Preparation of documents for the study process: fieldwork manuals, nursing and intervention measurements, educational leaflets and list of measurements.

• Written documentation: electronic and hard copies of the protocol, signed consent forms, and patient results stored under lock and key. All written information, including letters sent to patients is standardised in each of the centres.

• Training for those in charge of the standardisation of the study process.

• Training for nurses concerning the study characteristics and procedures, and, in particular, the quality of life interview to be given to the patients.

• Holding of regular meetings: with the coordinator of the study (daily) and with the principal investigator of each collaborating centre (weekly).

• Quarterly meetings and daily contact by email with the members of the EFICAR group and all the participating centres.

• Monthly production of progress reports.

### Ethical and legal aspects

This study complies with the Declaration of Helsinki and subsequent revisions as well as with correct clinical practice.

Data confidentiality: only the researchers involved with this study have access to data concerning the individuals who have agreed to participate in the study.

The following documents have been obtained:

• Approval of the Ethics Committee and the Spanish Personal Protection Act according to the Act 15/1999 and recent regulation (RD 1720/2007).

• Report from the research commission.

• "Understanding and commitment to collaborate from the services involved" signed by the legal representative of each Centre.

### Limitations

Despite the study not dealing with mortality and hospital admissions as a main measure, it is important to highlight that the HF-Action study has demonstrated that the effect of exercise on mortality is minimal. Given this, the functional capacity and quality of life are the most important aspects to improve in the rehabilitation of these patients.

Also, the structure of the study renders impossible blinding the participants and the interveners, however, blind outcome assessors will be used.

The monitoring of this intervention and data collection is complex, and therefore data will be collected using many relevant quality control processes, and standardization of the intervention will be assured. In addition, to avoid contamination of the control group, interveners will be trained, and a pilot study will also be carried out.

## Discussion

As well as being highly relevant, this study presents great challenges as mentioned earlier, in particular, the organisational challenges and that of guaranteeing the safety of the exercise programme for patients, since we are dealing with patients suffering with NYHA class II-IV heart failure.

Available scientific evidence, discussed below, enables us to guarantee that carrying out the EFICAR Project does not increase the risk of decompensation, cardiac events, hospital admission or death, but quite the opposite [27]. Safety throughout the project is based on the fact that all patients are going to benefit from the best standardised pharmacological treatment and health education. Indeed, their treatments will be more carefully monitored than conventional treatment, for either of the groups the patients will be assigned to: control or exercise. Specifically, prior to their designation to any of the aforementioned groups, patients must go through a process of therapy optimisation and no patient will take part in the study if they do not have optimal treatment or are not in a stable clinical condition.

### • Optimisation of regular care for the eligible patients prior to their inclusion in the study

At the start, every patient eligible to participate will be assessed in standarised nurse and medical appointments. Their functional and cognitive state, degree of dependence, clinical condition (symptoms and vital signs), body fluids, risk factors, health habits (including exercise in daily life), vaccination status, ECG, psychological, social and work conditions and quality of life will be determined. On the basis of this assessment, the issues to be tackled will be identified and educational and clinical objectives established, with standardisation of treatment and follow-up, monitoring trigger and worsening factors and concomitant diseases, setting criteria for referral to the cardiology unit and ensuring the absence of contraindications for exercise.

Subsequently, the nurse unit will undertake a planned programme of health education, promote self-care and standardised regular monitoring of the elements considered important in the initial assessment. The monitoring appointments will be scheduled weekly during the first month, every other week for the second and third months, and then monthly until the end of the study. In addition, extra appointments may be necessary for the monitoring of altered factors, guaranteeing the accessibility and protocolised care for decompensation and emergencies.

### • Risks of the intervention

Whether the exercise is effective or not, patients will not run additional risks, since exercise for patients with HF does not increase the number of clinical events or likelihood of death, but quite the opposite. We can estimate the risk of the intervention on the basis of some reviews and in the HF-Action study carried out with more than 2300 NYHA class II-IV individuals [16,28].

Smart et al. (2004) carried out a systematic review of 81 studies (30 randomised clinical trials) to assess the effect of exercise on mortality and morbidity of people with HF very similar to the patients participating in our study. No exercise-related deaths were reported during the 60,000 patient hours of physical exercise. The authors found evidence of a small reduction of the variable combining mortality and adverse events, and a possible beneficial effect on survival after carrying out the exercise. Thus, they concluded that those patients with HF who carried out an exercise programme have some type of clinical event every 3700 hours, a figure lower than that for those who do not exercise.

Reinforcing these conclusions, the recent study HF-Action [16,17], the largest clinical trial undertaken with 2331 people with NYHA class II-IV HF, to evaluate the effect of aerobic exercise on mortality and number of hospital admissions, has demonstrated that physical exercise interventions are safe for people suffering from HF. This study concludes that exercise even has a small beneficial effect (a reduction in mortality and hospital admissions of 11%). As the worst case scenario could be expected to be one clinical event for every 3700 patient hours of exercise [28], the probability of a clinical event occurring in each centre can be estimated at 39%. It can be interpreted from this that with ten collaborating centres, we should expect to have no clinical event in six of these centres by the end of the Project. In the case of mortality, under the worst case scenario, the probability of having one death in each of the collaborating centre is estimated to be 1 per 1000.

### • Safety system for cardiac emergencies in health centres

Carrying out this study will be also present an opportunity to test the system for action in the each of the health centres should a cardiac emergency occur, regardless of the fact that this study will not increase their occurrence, quite the contrary.

In order to guarantee safety in cardiac emergencies, regardless of the implementation of EFICAR Project, the actions described in the 'Additional file 1' are recommended.

### • Safety Committee

The data will be provisionally analysed by an independent committee to monitor safety. This committee will be composed of people who are independent of the body in charge of the management of the study and of the researchers and will be blind to the assignment of patients to the comparison groups. It will define the indicators related to mortality, hospital admissions and decompensations, and will have independent access to the necessary data. It will review the data of study every quarter and will make recommendations to the team in charge of the project with respect to the continuation or interruption of the study.

## Competing interests

The authors declare that they have no competing interests.

## Authors' contributions

Conception of the idea for the study: Gonzalo Grandes and Ana Zuazagoitia. Development of the protocol, organization and funding: Ana Zuazagoitia, Gonzalo Grandes, Jesus Torcal, Iñaki Lekuona, Pilar Echevarria, Manuel Angel Gómez, MMar Domingo, M^a ^del Mar de la Torre, Jose Ignacio Ramírez, Imanol Montoya, Juana Oyanguren and Ricardo Ortega-Sánchez Pinilla. Cardiological clinical committee responsible for the elegible criteria, safety and validity of the outcomes: Iñaki Lekuona, Santi Palomar, Jose Antonio Alarcón, Andrés Grau Sepúlveda, Josep Lupón, Ignacio Santos, Maximiliano Diego, Ignacio Cruz, Javier Moreiras, Candido Martín Luengo, Pedro Pavón, M^a ^del Mar de la Torre Carpente. Primary care clinical committee responsible for the intervention programme, treatment, adherence and monitoring: Ana Zuazagoitia, Gonzalo Grandes, Jesus Torcal, Iñaki Lekuona, Pilar Echevarria, Manuel Angel Gómez, M^a ^del Mar Domingo, M^a ^del Mar de la Torre, Jose Ignacio Ramírez, Imanol Montoya, Juana Oyanguren and Ricardo Ortega-Sánchez Pinilla + grupo EFICAR. Coordinating centre: Ana Zuazagoitia, Gonzalo Grandes and Imanol Montoya of the Primary Care Research Unit of Bizkaia. Writing of the manuscript: Ana Zuazagoitia and Gonzalo Grandes. All the authors have read the draft critically, to make contributions, and have approved the final text.

## Pre-publication history

The pre-publication history for this paper can be accessed here:

http://www.biomedcentral.com/1471-2458/10/33/prepub

## Supplementary Material

Additional file 1 **Safety system for cardiac emergencies in the health**. This file contains the step and actions that are needed to follow in order to guarantee safety in cardiac emergencies, regardless of the implementation of EFICAR Project. Click here for file
