# Supplementary material for: Rationale and design of a randomised controlled trial evaluating the effectiveness of an exercise program to improve the quality of life of patients with heart failure in primary care: The EFICAR study protocol
Source: BMC Public Health. 2010 Jan 25;10:33. doi: 10.1186/1471-2458-10-33 (PMC2835681; doi:10.1186/1471-2458-10-33)
Supplement: Additional file 1 — Safety system for cardiac emergencies in the health. This file contains the step and actions that are needed to follow in order to guarantee safety in cardiac emergencies, regardless of the implementation of EFICAR Project. [file 1471-2458-10-33-S1.DOC]

**Appendix 1**.

**Safety system for cardiac emergencies in the health**

- Protocols agreed between primary care-cardiology will be established for key cardiac emergencies. These will include at least Cardiopulmonary Resuscitation (CPR), Acute Coronary Syndrome (ACS), Acute Pulmonary Oedema (APO), bradiarrhythmias, tachyarrhythmias, hypertensive emergencies. Said protocols will be signed by both parties.
- There will be training courses for all the members of the research team and for enough health professionals from the centres to cover absences.
- Said professionals will be assessed on the application of the protocols; they must pass and obtain the certification in order to take part in the project. In the case some fail to obtain the certification, the training process will continue until they pass.
- The people involved in the assessment will be cardiologists or primary care doctors and nurses previously tested by the cardiology unit in relation to implementation of these protocols.
- The professionals who should attend an emergency must be identified and available at any time within the health centre.
- The action that nursing staff may and should take with respect to the implementation of the protocols until the arrival of the doctor will be specified in writing and signed. In no case would this imply the non-attendance of the medical professionals.
- The nursing staff will be trained in the recognition of “alarm signs and symptoms”, in the recognition of at least the following ECG changes: ST segment elevation/depression in ACS, second and third degree AV blocks (atrioventricular block), sinusal rhythm, AFA (atrial fibrillation arrhythmia), regular/irregular narrow QRS complex (tachycardia), regular/irregular wide QRS complex (tachycardia) and ventricular fibrillation (VF).
- There will be standardised guidelines for not starting physical exercise or stopping in accordance with the clinical status of the patient.
- The medical history, allergies, current medication and basal ECG will available at all times in the place where the exercise takes place.
- Patients will be asked to show their self-care booklets before starting exercise.
- There will be emergency protocols and their effectiveness will be tested, as appropriate, by at least three simulations with no warning, evaluating the response times.
- Whenever there is an emergency, the attending team will make an assessment and submit a report to the principal investigator, who in turn assesses the incident and informs the other researchers. The patient’s history, a description of what occurred and the guidelines applied are detailed. This is compared with the protocol. Measures for improvement in the case of similar situations are proposed and the probable cause is indicated if known.
- There will be a minimum emergency kit in all the places where the exercise activity will be carried out by HF patients. This will include at least: one crash trolley with defibrillator for synchronised and non-synchronised cardioversion as well as medication and emergency kit, oxygen, suction units, pulse oximeter, ECG, blood pressure machine, glucometer, protocol and log sheets. The stock of these supplies is checked regularly, with defined responsibility (ie signed and dated by the person who checks the trolley).
- Ways will be established, using mobile telephones, to alert the rest of the emergency team.
- There will be meetings of the research team expressly for the purpose of suggesting complementary measures and reviewing existing ones.
- In this way it is proposed that the emergency process complies with the ISO quality norms.
